# Clinical trial simulation to evaluate power to compare the antiviral effectiveness of two hepatitis C protease inhibitors using nonlinear mixed effect models: a viral kinetic approach

**DOI:** 10.1186/1471-2288-13-60

**Published:** 2013-04-25

**Authors:** Cédric Laouénan, Jeremie Guedj, France Mentré

**Affiliations:** 1 INSERM, UMR 738, Université Paris Diderot, Sorbonne Paris Cité, Paris F-75018, France; 2 AP-HP, Hôpital Bichat, Service de Biostatistique, Paris F-75018, France

**Keywords:** Hepatitis C virus, Non-linear mixed effect models, Early viral kinetics, Protease inhibitor, Mathematical modeling, Direct-acting antiviral agents

## Abstract

**Background:**

Models of hepatitis C virus (HCV) kinetics are increasingly used to estimate and to compare *in vivo* drug’s antiviral effectiveness of new potent anti-HCV agents. Viral kinetic parameters can be estimated using non-linear mixed effect models (NLMEM). Here we aimed to evaluate the performance of this approach to precisely estimate the parameters and to evaluate the type I errors and the power of the Wald test to compare the antiviral effectiveness between two treatment groups when data are sparse and/or a large proportion of viral load (VL) are below the limit of detection (BLD).

**Methods:**

We performed a clinical trial simulation assuming two treatment groups with different levels of antiviral effectiveness. We evaluated the precision and the accuracy of parameter estimates obtained on 500 replication of this trial using the stochastic approximation expectation-approximation algorithm which appropriately handles BLD data. Next we evaluated the type I error and the power of the Wald test to assess a difference of antiviral effectiveness between the two groups. Standard error of the parameters and Wald test property were evaluated according to the number of patients, the number of samples per patient and the expected difference in antiviral effectiveness.

**Results:**

NLMEM provided precise and accurate estimates for both the fixed effects and the inter-individual variance parameters even with sparse data and large proportion of BLD data. However Wald test with small number of patients and lack of information due to BLD resulted in an inflation of the type I error as compared to the results obtained when no limit of detection of VL was considered. The corrected power of the test was very high and largely outperformed what can be obtained with empirical comparison of the mean VL decline using Wilcoxon test.

**Conclusion:**

This simulation study shows the benefit of viral kinetic models analyzed with NLMEM over empirical approaches used in most clinical studies. When designing a viral kinetic study, our results indicate that the enrollment of a large number of patients is to be preferred to small population sample with frequent assessments of VL.

## Background

Chronic infection with Hepatitis C Virus (HCV) affects 130–200 million people worldwide [1]. It is the leading cause of cirrhosis, liver cancer and liver transplants which result in 350,000 deaths worldwide [2]. HCV is divided into 6 genotypes, with genotype 1 being the hardest to treat and the most prevalent in Western countries. The goal of treatment is to achieve a sustained virologic response (SVR), marker of viral eradication, assessed by a viral load HCV RNA (VL) below the limit of detection (LOD) six months after cessation of therapy. Until 2011, the only available treatment was based on weekly injections of pegylated interferon (peg-IFN) and daily oral ribavirin (RBV) during 48 weeks, with SVR rate lower than 50% in treatment-naïve HCV genotype 1 patients [3].

In 2011, the approval of two protease inhibitors (PI), telaprevir and boceprevir, in combination with peg-IFN/RBV (triple therapy), marked a milestone for anti-HCV therapy with SVR rates larger than 70% in treatment-naïve HCV genotype 1 patients [4,5]. Dozens of compounds targeting different viral proteins are currently in different stages of clinical trials, raising the expectation that several IFN-free regimens might be available in the coming years.

Viral kinetic modeling aims at characterizing the main mechanisms that govern the virologic response to treatment using mathematical models. Following the recommendations of the Food and Drug Administration [6], this approach has been increasingly used in phase 1/2 of clinical development to estimate viral kinetic parameters and to evaluate drug antiviral effectiveness *in vivo*[7,8]. Parameter estimation is often achieved using non-linear mixed effect models (NLMEM) [9]. The popularity of this approach is due to the fact that it optimizes the information available by borrowing strength from the whole sample to provide precise estimation of the parameters, including covariate effects [10-12]. Moreover it naturally accounts for the information brought by VL data below the limit of detection (BLD) and reduces the bias in parameter estimation as compared to empirical approaches where BLD data are ignored or assigned to half the LOD [10,13,14].

So far, viral kinetic models and NLMEM have mostly been used in phase 1/2 clinical trials with large number of patients and/or frequent assessment of VL data within each patient. However in most clinical trials, in particular when they are not sponsored by the industry, it is not possible to hospitalize patients and to get frequent viral load samples. In this challenging context, the capacity of NLMEM to precisely estimate viral kinetic parameters is not known. In particular the performance of tests used to assess the effect of a covariate which have good asymptotic properties (Wald test, likelihood ratio test or score test) is not warranted when one is far from the asymptotic conditions. For instance an inflation of the type I error has been reported in another clinical context where data were sparse [15]. With the new potent triple therapies against HCV the amount of information available may also be limited by the fact that a large proportion of VL data are below LOD.

Here our goal was to evaluate the capacity of NLMEM to precisely estimate the parameters of viral kinetic models when there is a large proportion of BLD data and a limited number of data per patient. In particular we aimed to evaluate by simulation the type I errors and the power of the Wald test to compare the antiviral effectiveness of two groups receiving different triple therapies (noted PI-A and PI-B in the following). Parameter estimation and Wald test property were evaluated according to the number of patients, the number of samples per patient and the expected difference in antiviral effectiveness between the two treatment groups.

## Methods

### Viral kinetic model

We used the standard biphasic model of HCV kinetics defined by the following set of differential equations [16]:

(1) $$\left\{\begin{array}{l} \frac{\mathrm{dI}}{\mathrm{dt}}=\mathrm{bVT}-\mathrm{\delta I}\\ \frac{\mathrm{dV}}{\mathrm{dt}}=p\left(1-\epsilon \right)I-\mathrm{cV} \end{array}\right)$$

where *T* represents the density of target cells that can be infected by virus measured as HCV RNA (*V*), with rate constant *b*. In the model, infected cells (*I*) die or lose their infected state with rate constant *δ* and produce virions at constant rate *p* per cell. Virions are assumed to be cleared with rate constant *c*. As it is done usually when considering short term VL data we assumed that the target cell level is constant throughout the study period and remains at its pre-treatment steady state value *T*_*0*_* = cδ/pb*[17,18].

Treatment is assumed to reduce the average rate of viral production per cell from *p* to *p*(1–*ϵ*), where *ϵ* represents the constant drug effectiveness, *i.e.*, *ϵ* = 0.990 implying the drug is 99% effective in blocking viral production. If all parameters including treatment effectiveness are constant over time this model predicts that VL will fall in a biphasic manner [16], with a rapid first phase of viral decline with rate approximately equal to *c* lasting for a couple of days and with the magnitude viral decline depending on *ϵ*, and a second slower but persistent second phase of viral decline with rate *ϵδ*. Hence, for potent therapies for which *ϵ* is close to 1, the second-phase slope will be approximately *δ*.

Lastly mathematical analysis shows that if *ϵ* is constant, *p* and *b* do not intervene in the VL equation and thus where ignored in the following without loss of generality [19].

### Statistical model

We assumed an additive error (σ) on the log_10_ of the VL observations, *i.e.*, the observed data *y*_*ij*_ for patient *i* at time *t*_*ij*_ is given by:

(2) $$y_{\mathrm{ij}}=f\left(\phi_{i},\;t_{\mathrm{ij}}\right)+e_{\mathrm{ij}},$$

(3) $$e_{i}∼N\left(0,\;\sigma^{2}\right),$$

(4) $$h\left(\phi_{i}\right)=\mu +\beta T_{i}+\eta_{i}\;\mathrm{with}\;\eta_{i}∼N\left(0,\;\Omega \right),$$

where:

— *f* is the non-linear model,

— *Φ*_*i*_ is the vector of individual parameters of length p where p is the number of parameters,

— *e*_*ij*_ is the residual error assumed to follow a normal distribution with mean 0 and variance *σ*^2^,

— *h* is the transformation of the vector of parameters that make them normally distributed,

— *μ* is the vector of fixed effects,

— *β* is the vector of coefficient of the only covariate studied *i.e.* the difference of effectiveness between PI-A and PI-B (with *T*_*i*_ = 0 if treatment is PI-A and *T*_*i*_ = 1 if treatment is PI-B),

— *η*_*i*_ is the vector of random effects independent of *e*_*i*_, and are supposed to be independent, with diagonal variance-covariance matrix $\Omega =\mathrm{diag}\;\left(\omega_{1}^{2},\ldots ,\omega_{p}^{2}\right)$.

It is assumed that *h* is the logarithm transformation for *V*_*0*_, δ and *c* and the logit transformation for *ϵ.*

### Parameter values

Mean parameter values, inter-individual standard deviations (*ω*) and standard deviation of residual error, σ, for patients treated with PI-A were assumed to be similar to those found in phase 1 of clinical trials with telaprevir at steady state [7] (Table 1). We assumed that PI-A immediately reached its steady state level of effectiveness, with mean *ϵ*^A^ = 0.999. Similar parameter distribution was assumed in patients treated with PI-B, except for the mean antiviral effectiveness of PI-B, *ϵ*^B^. We considered several values for *ϵ*^B^ equal to 0.999, 0.998, 0.995 and 0.990, corresponding to a similar, 2-fold, 5-fold and 10-fold lower levels in the blocking of viral production than PI-A, respectively. The LOD was fixed to 12 IU/mL [20].

**Table 1:** **Value, distribution and inter-individual standard deviation of population parameters from data with patients treated by monotherapy of PI-A **[7]

	***V***_***0***_**(IU/mL)**	***c*(day**^**-1**^**)**	***δ*(day**^**-1**^**)**	***ϵ***	**σ (log**_**10**_**IU/mL)**
Fixed effect	2.68 10^6^	13.4	0.58	0.999	0.19
Transformation	lognormal	lognormal	lognormal	logistic-normal	-
Inter-individual standard deviation (ω)	1.09	0.25	0.25	0.61	-

### Clinical trial simulation

**Figure 1:**
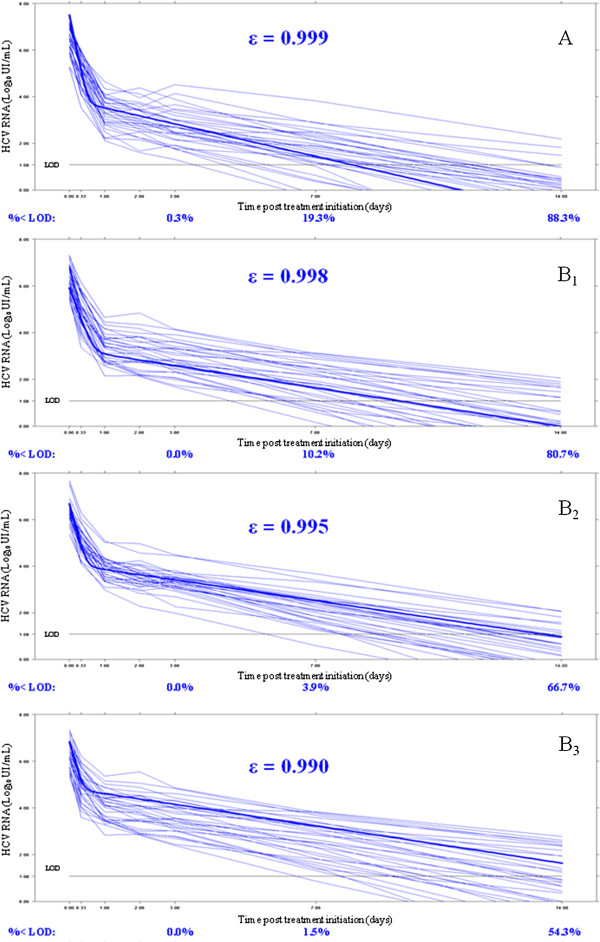
**Time course of log**_**10 **_**HCV RNA after treatment initiation for one simulated dataset with one design (N = 30 patients per PI and n = 7 viral load measurements). A**: assuming ϵ = 0.999; **B1**: assuming ϵ = 0.998; **B2**: assuming ϵ = 0.995; **B3**: assuming ϵ = 0.990. In bold, the mean curves predicted by the mean parameters of the model. LOD: limit of detection = log_10_(12) ≈ 1.08 log_10_ UI/mL; % < LOD: percentage of patients bellow the LOD at day 3, 7 and 14 estimated from 500 simulated datasets. The mean 14 days log drop were 6.56 log_10_ IU/mL with *ϵ* = 0.999, 6.25 log_10_ IU/mL with *ϵ* = 0.998, 5.85 log_10_ IU/mL with *ϵ* = 0.995 and 5.52 log_10_ IU/mL with *ϵ* = 0.990.

We considered different designs in real-life setting, *i.e.,* with a limited number of VL measurements per patient. Two schedules for the VL assessments were considered, called “7 VL” and “5 VL” in the following. “7 VL” had seven VL measurements at days 0, 0.33, 1, 2, 3, 7 and 14 whereas “5 VL” was sparser and did not have the early measurements at days 0.33 and 1 that are often difficult to obtain in clinical practice. Then different scenarios were considered according to the number of VL measurements (n) and the number of patients per group of treatment (N). In order to have designs that could be easily compared, we considered different designs with 5 VL or 7 VL but constant total numbers of observations per group n_tot_ = N×n. We considered small sample size with: n_tot_ = 50 (N = 10 and n = 5) and n_tot_ = 70 (N = 10 and n = 7 or N = 14 and n = 5), middle sample size with: n_tot_ = 100 (N = 20 and n = 5) and n_tot_ = 140 (N = 20 and n = 7 or N = 28 and n = 5) and larger sample size with: n_tot_ = 150 (N = 30 and n = 5) and n_tot_ = 210 (N = 30 and n = 7 or N = 42 and n = 5). For each scenario, K = 500 dataset were generated using R software version 2.15.0 (R foundation for Statistical Computing, Vienna, Austria). Examples of simulated dataset with the design N = 30 and n = 7 and the different levels of antiviral effectiveness considered with the percentage of patients below the LOD at day 3, 7 and 14 is shown in Figure 1.

### Parameter estimation

Data of each simulated trial were analyzed using MONOLIX version 4.2 (http://www.lixoft.eu/monolix/product-monolix-overview/) [21], a software devoted to maximum likelihood estimation of parameters in NLMEM using an extension of the stochastic approximation expectation-approximation (SAEM) algorithm [22,23]. Of note one advantage of maximum likelihood estimation (noted “ML” in the following) is that it takes into account the information brought by BLD data [10]. We compared accuracy and precision of parameter estimations obtained with those that would be obtained if all data were observed with no BLD data at all (referred as “all data”).

For each scenario, relative estimation errors REE $\left({\hat{\theta }}_{k}\right),k=1,\ldots ,K$ were computed as shown in equation (5), where ${\hat{\theta }}_{k}$ is the parameter estimated for the k^th^ replicate and θ^*^ the true parameter value used to generate the data.

(5) $$\mathrm{REE}\left({\hat{\theta }}_{k}\right)=\frac{{\hat{\theta }}_{k}-\theta^{*}}{\theta^{*}}\times 100$$

Each REE_k_ was expressed in percent. We plotted the boxplot of the REE with the 10% and 90% percentiles. Then, from the REE, the relative bias (RB) and the relative root mean square error (RRMSE) were computed as shown in equation (6) and (7).

(6) $$\mathrm{RB}\left({\hat{\theta }}_{k}\right)=\frac{1}{k}\sum_{k=1}^{K}\mathrm{REE}\left({\hat{\theta }}_{k}\right)$$

(7) $$\mathrm{RRMSE}\left({\hat{\theta }}_{k}\right)=\sqrt{\frac{1}{k}\sum_{k=1}^{K}\mathrm{REE}{\left({\hat{\theta }}_{k}\right)}^{2}}$$

The accuracy and the precision of parameter estimates were evaluated using RB and RRMSE, respectively.

### Detection of a difference in antiviral effectiveness

We analyzed for each scenario the ability to detect a difference of antiviral effectiveness between the two PIs. Given that treatment antiviral effectiveness was estimated on a logistic scale we defined accordingly the difference of effectiveness between the two PIs, *β*, as *β* = *logit*(*ϵ*^*A*^) − *logit*(*ϵ*^*B*^). For each simulated dataset, the estimated value of $\beta ,\hat{\beta }$, was obtained along with its (estimated) standard error, $SE_{\hat{\beta }}$. Then the Wald test statistics given by $\frac{\hat{\beta }}{SE_{\hat{\beta }}}$ was calculated and the null hypothesis “H_0_: *β* = 0” was rejected at the level of 5% if $\left|\frac{\hat{\beta }}{SE_{\hat{\beta }}}\right|>1.96$. Thus in scenarios where *ϵ*^A^*= ϵ*^B^ = 0.999 and therefore *β* = 0, the type I error was given by the proportion of dataset among the 500 simulated that led to reject H_0_. Similarly the power to detect a difference in treatment antiviral effectiveness was calculated in scenarios where *β* ≠ 0 and was given by the proportion of datasets among the 500 simulated that led to reject H_0._ With *ϵ*^A^ = 0.999 and with values of *ϵ*^B^ equal to 0.998, 0.995 and 0.990, *β* were equal to 0.7, 1.6 and 2.3, respectively. Type I error and power were evaluated with all designs describe above and consistent with previous analysis [15,24], we expect an inflation of the type I error with the Wald test. To ensure a type I error of 5%, we define for each design a correction threshold as the 5^th^ percentile of the distribution of the p-values of the test under H_0_ for the 500 simulated dataset. Then we used that corrected threshold as a limit of significance in the evaluation of the tests under H_1_ to compute the corrected power [15,25]. Furthermore we evaluated type I error with 2 larger sample size to approach asymptotic conditions with: n_tot_ = 350 (N = 50 and n = 7) and n_tot_ = 700 (N = 100 and n = 7). Lastly, the power to detect a difference in treatment effectiveness between the two PIs was compared with the one obtained by standard empirical approaches where the difference in viral decline at day 14 between two treatments is tested by a non parametric two-sided Wilcoxon test.

## Results

### Parameter estimation

First we evaluated the impact of having a large proportion of BLD data on the precision of parameter estimates. Proportions of BLD data were equal to 19.3% and 88.3% at days 7 and 14 with ϵ = 0.999, 10.2% and 80.7% with ϵ = 0.998, 3.9% and 66.7% with ϵ = 0.995, 1.5% and 54.3% with ϵ = 0.990, respectively (Figure 1). For that purpose we compared the parameters estimation with all data or ML with the design N = 30 and n = 7 and assuming a lower effectiveness for PI-B than PI-A (*ϵ*^A^ = 0.999 vs *ϵ*^B^ = 0.990).

**Figure 2:**
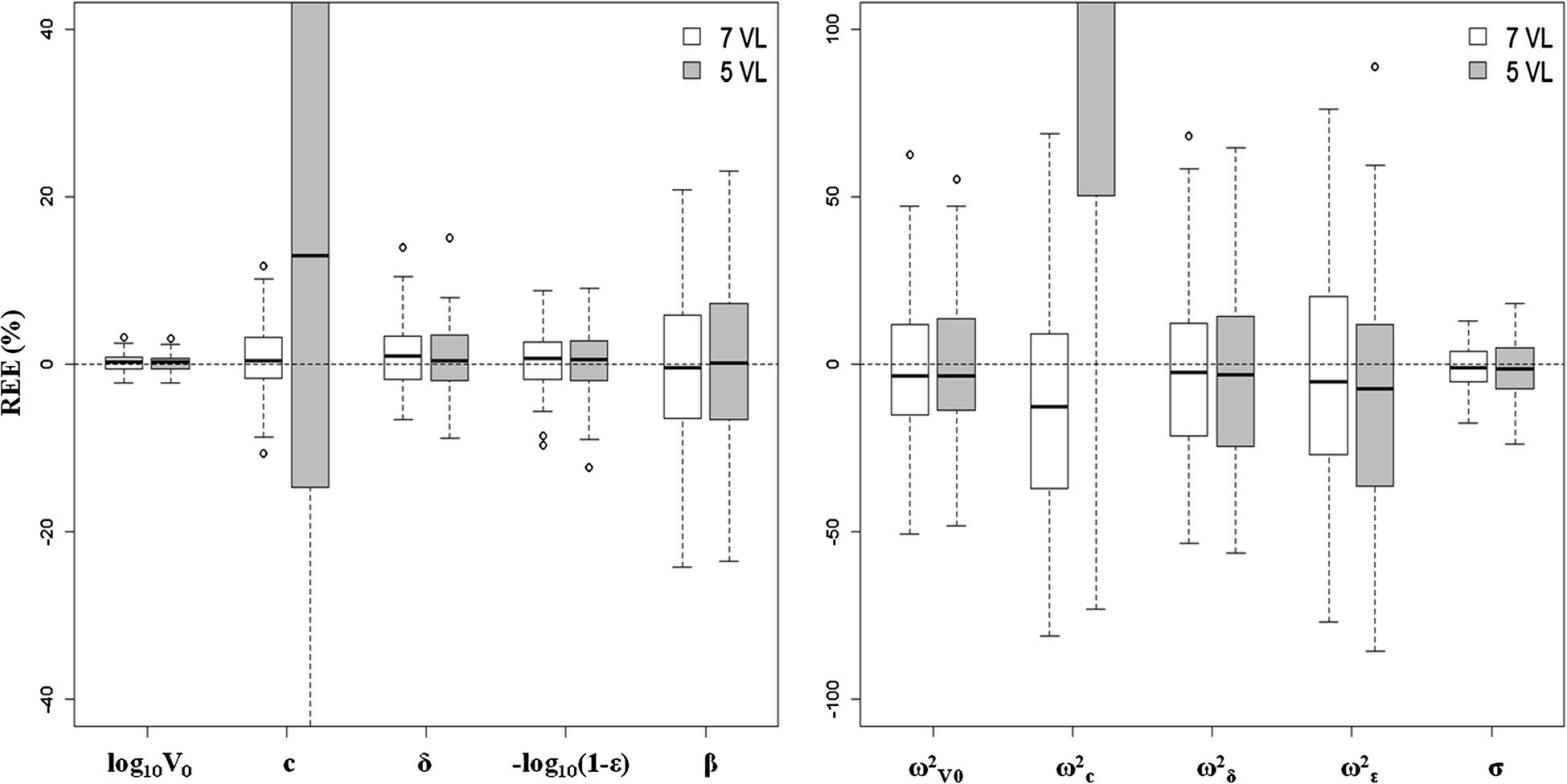
**Boxplot of the relative estimation errors (REE) of the estimated parameters evaluated from 500 simulated datasets.** Parameters were simulated assuming ϵ^A^ = 0.999, a limit of detection (“ML data”) and ϵ^B^ = 0.990 (*β* = 2.3*)*, with N = 30 patients per PI and with n = 7 viral load (VL) measurements (in white) or n = 5 VL (sparse initial design in gray). On the left: fixed effects and on the right: inter-individual variances (ω²) and residual error (σ).

Assuming all data, *i.e.,* all data can be observed and there is no LOD, all the parameter estimates had a very small RB lower than 1% and 11% for the fixed effects and the inter-individual variance parameters, respectively. Similarly, the RRMSE were lower than 10% and 33% for the fixed effects and the inter-individual variance parameters, respectively (Table 2). Yet, the precision of both the fixed effect and inter-individual variance parameters were very close to those found with the ML uncensored data (Table 2), showing the relevance of maximum likelihood in the handling of censored data. Similar results were obtained when comparing the distribution of the REE from the 500 simulated datasets (Figure 2). Equally good performance was obtained when considering sparser sampling design with n = 5 VL measurements per patient except for the viral clearance rate, *c* (Table 2 and Figure 2) [11].

**Table 2:** Relative bias (RB) (%) and relative root mean square error (RRMSE) (%) of the estimated parameters evaluated from 500 simulated datasets

	**All data (n = 7 VL)**		**ML (n = 7 VL)**		**ML (n = 5 VL)**	
	**RB (%)**	**RRMSE (%)**	**RB (%)**	**RRMSE (%)**	**RB (%)**	**RRMSE (%)**
log_10_(*V*_*0*_) (IU/mL)	0.1	1.0	0.2	1.0	0.1	1.0
*c* (day^-1^)	1.0	4.3	0.6	4.1	34.1	78.8
*δ* (day^-1^)	0.2	3.2	0.8	3.7	0.6	3.7
-log_10_(1-*ϵ*)	0.1	3.2	0.4	3.1	0.3	3.6
*β*	0.4	8.5	−0.5	8.5	0.4	9.9
_ω_^2^_vo_	−0.4	18.9	−1.0	19.0	−0.5	19.3
_ω_^2^_c_	−4.6	31.5	−10.9	32.9	236.6	358.3
_ω_^2^_δ_	−3.0	19.8	−2.3	24.5	−2.4	24.9
_ω_^2^_ϵ_	−2.6	32.2	−4.5	32.0	−9.9	36.3
*σ*	−0.03	5.3	−0.7	6.2	−1.3	8.0

Parameters were simulated with N = 30 patients per group assuming *ϵ*^A^ = 0.999 and *ϵ*^B^ = 0.990, no limit of detection (“All data”) or a limit of detection at 12 IU/mL (“ML”), and with n = 7 or 5 viral load (VL) measurements per patient.

### Type I error of the Wald test

**Figure 3:**
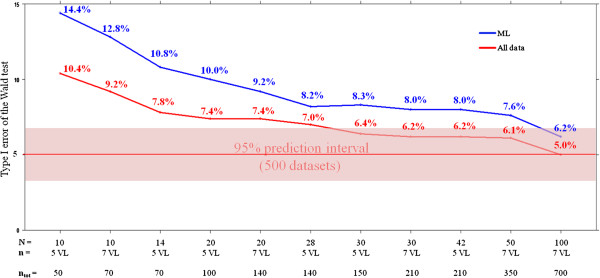
**Evolution of the type I error of the Wald test according to the study design.** Assuming ϵ^A^ = ϵ^B^ = 0.999, no limit of detection (“All data”, red line) or a limit of detection at 12 IU/mL (“ML”, blue line). N: number of patients per group of treatment; n: number of viral load measurements per patient; n_tot_: total numbers of observations per group of treatment.

Next we evaluated the type I error of the Wald test according to different designs and assuming *ϵ*^A^*= ϵ*^B^ = 0.999 (Figure 3). A type I error of 14.4% was found with ML data, n = 5 VL and N = 10 showing that the asymptotic conditions under which the Wald test is valid were not met with this design. In fact a minimal number of observations per group n_tot_ = 140 was necessary in order to achieve a type I error less than 10% and n_tot_ = 700 (N = 100 and n = 7) were needed for type I error to be in the 95% prediction interval around 5% [3.1%; 6.9%] with ML data. Of note, for a given value of N, the number of VL measurements did not substantially change the type I error (Figure 3) and increasing the number of patients N was more beneficial than having more frequent VL assessments within each patient. For instance the type I error was lower with the design N = 14 and n = 5 than with the design N = 10 and n = 7 (Figure 3) although the total number of observation per patient n_tot_ was the same and equal to 70. In addition to the influence of the number of patients, the type I error was also deteriorated by the presence of a large proportion of BLD data, even if they were taken into account appropriately. Indeed type I errors were consistently smaller when assuming that there was no LOD (“all data”). As shown in Figure 3, the type I error was equal to 10.4% with all data, N = 10 and n = 5 compared to 14.4% with ML data.

### Power to detect a difference in antiviral effectiveness

Next, we evaluated the power to detect a difference of effectiveness between the two PIs assuming *ϵ*^A^ = 0.999 and lower values of *ϵ*^B^ equal to 0.998, 0.995 and 0.990 with different designs. Except when both effectiveness were close (*i.e.*, *ϵ*^B^ = 0.998), the power of the Wald test, corrected or not (see methods), was larger than 95%, regardless of the number of sampling VL measurements and the number of patients per PI (Table 3). As found for the type I error, increasing the number of patients N was more beneficial than having more frequent VL assessments within each patient. For instance power was higher with the design N = 28 and n = 5 than with the design N = 20 and n = 7 (Table 3) although the total number of observation per patient n_tot_ was the same and equal to 140.

**Table 3:** **Power (%) to detect a difference of effectiveness between PI-A and PI-B according to the study design and the effectiveness of PI-B (*ϵ***^**B**^**), assuming *ϵ***^**A **^**= 0.999 and a limit of detection (“ML data”)**

	***ϵ***^**B**^	**0.998**	**0.995**	**0.990**	**0.998**	**0.995**	**0.990**	**0.998**	**0.995**	**0.990**
Small sample size	Design*	N = 10 and n = 7			N = 14 and n = 5			N = 10 and n = 5		
		n_tot_ = 70			n_tot_ = 70			n_tot_ =50		
	Wald test (uncorrected)	62.2	99.8	100	61.8	100	100	55.2	98.8	100
	Wald test (corrected)	44.2	98.4	100	50.4	100	100	35.8	95.8	100
	Wilcoxon test	6.6	11.2	26.8	4.4	15.6	39.0	6.6	11.2	26.8
	Design*	N = 20 and n = 7			N = 28 and n = 5			N = 20 and n = 5		
		n_tot_ = 140			n_tot_ = 140			n_tot_ = 100		
Middle sample size	Wald test (uncorrected)	83.4	100	100	86.8	100	100	77.8	100	100
	Wald test (corrected)	69.0	100	100	78.0	100	100	58.8	100	100
	Wilcoxon test	7.0	23.0	50.4	6.8	30.4	64.6	7.0	23.0	50.4
Large sample size	Design*	N = 30 and n = 7			N = 42 and n = 5			N = 30 and n = 5		
		n_tot_ = 210			n_tot_ = 210			n_tot_ = 150		
	Wald test (uncorrected)	94.0	100	100	86.8	100	100	89.4	100	100
	Wald test (corrected)	89.2	100	100	82.6	100	100	82.6	100	100
	Wilcoxon test	7.4	31.0	67.0	9.2	43.8	85.0	7.4	31.0	67.0

* N: number of patients per group of treatment; n: number of viral load measurements per patient; n_tot_: total numbers of observations per group of treatment.

Lastly, we compared these results with the power achieved by comparing the mean viral decline at day 14 between both groups using a Wilcoxon test. The power of Wilcoxon test was on average lower of 44% as compared to the one achieved with viral kinetic model and NLMEM with *ϵ*^B^ = 0.995 or 0.998 (Table 3). Even when PI-B’s and PI-A’s antiviral effectivenesses were assumed to be 0.990 and 0.999, respectively, corresponding to a 10-fold difference in the viral production under treatment, the power of the Wilcoxon test was only equal to 67% with N = 30, compare to 100% with the Wald test (Table 3). It should be noted that the type I error of the Wilcoxon test, which is a non parametric test, was not inflated (5.2% with the design N = 10 for example).

## Discussion

The goal of this study was to evaluate the capacity of NLMEM to provide precise and accurate estimates of viral kinetic parameters when only sparse data with a large proportion of BLD data are available. In particular we aimed to evaluate the ability of this approach to correctly reject or not the null hypothesis of equal treatment effectiveness when two groups with different antiviral strategies are compared.

Our results showed that NLMEM provide very precise and accurate estimates for both the fixed effects and the inter-individual variance parameters, even when only 5 data points (at days 0, 2, 3, 7 and 14) were available within each patient. This allowed circumventing the need for intensive VL sampling measurements at treatment initiation, which are difficult to obtain in current clinical practice. Of note the viral clearance rate, *c* and its associated variability ω_c_, were poorly estimated in this sparse initial sampling. However this parameter is mostly involved in the initial rate of viral decline and thus a poor estimation of *c* did not substantially deteriorate the estimation of the other parameters (Table 2).

By comparing the results obtained with and without a LOD for VL, we demonstrated that maximum likelihood appropriately handle BLD, consistent with results found previously [10]. The conclusion was somewhat different when considering the outcome of Wald test for comparing antiviral effectiveness. In this case the lack of information due to BLD contributed to an inflation of the type I error as compared to the results obtained with no LOD of VL, suggesting that the development of real-time PCR assays with lower LOD may improve the estimation of viral kinetic parameters. Interestingly, even when there was no LOD of VL, we still found that the type I error was inflated when the number of observations n_tot_ was lower than 140. This suggests that the outcome of Wald test should be taken with caution when the number of patients is low and in that case we suggested to use a threshold correction for the Wald test to limit the impact of this inflation. Here we used an empirical threshold correction but other corrections exist such as the Galland correction or the permutation test [15]. On the other hand the power of the Wald test (corrected or not) was found to be very high, especially when compared with that obtained using a Wilcoxon test on the mean viral decline at day 14. This result clearly shows the benefit of viral kinetic analyzed with NLMEM over empirical approaches done in most clinical studies. Although better results may be obtained by comparing the viral decline at earlier time points (such as day 2 or 7) the power of the Wilcoxon test remained lower than those achieved by modeling approach (not shown). Consistent with results found elsewhere, the power increases when the number of observations per patient increases and was much less sensitive to the number of measurements within each patient [26]. From a clinical standpoint this finding indicates that the enrollment of a large population of study is to be preferred to small population sample with frequent assessments of VL.

We focused here on the properties of the Wald test and further studies would be needed to study how these results apply to other tests that require more computation time, such as likelihood ratio tests (LRT) or score test. Interestingly previous simulation studies using the SAEM algorithm in MONOLIX showed that the outcomes of these tests were largely comparable [15]. Of note this result may not hold when other estimation methods are used and for instance the outcomes of Wald test and LRT were found to be different when using the FOCE-I algorithm in NONMEM version 7 [15]. Indeed the Wald test had a lower power than LRT with FOCE-I, which was probably due to the poor estimation of the standard error of the covariate effect [25]. The advantage of the Wald test is that results are immediately obtained and do not require to compute the likelihood or its derivatives, as done for the LRT and the score test. Computation time needed by simulations could be largely reduced by using information theory and approximations to derive Fisher information matrix. For instance the software PFIM uses a first order approximation of the likelihood and, under this approximation, an analytical form of the Fisher matrix can be obtained [27]. Thus the expected variance of viral kinetic parameters could be obtained without the intensive simulations done here. Although such approximations worked well even with limited number of patients [11], it does not take into account BLD data and hence could underestimate the standard error when a large proportion of data are BLD. It should be noted that optimal design theory predicts that an increase of variances in random effect may deteriorate the precision of parameter estimates and the power of the Wald test. However this possibility was not investigated in this study where the inter-individual variance parameters were fixed.

Here we focused on the comparison of treatment antiviral effectiveness in the first two weeks of treatment. On this short time scale the standard biphasic model of viral kinetics has been shown to provide a good fit to the data [7,16]. However more complex models may be needed to fit long-term VL data, such as models that relax the assumption of constant target cells and/or account for the emergence of treatment resistant viruses [28,29]. Moreover viral decline during PI therapy is faster than what is observed with IFN-based therapy [30]. This feature is captured in the standard biphasic model by assuming that PIs lead to an enhancement of the treatment effectiveness, *ϵ*, and of the clearance rate of infected cells, *δ*[7,29,31]. Consistent with this observation we set here large mean values for both ϵ and *δ,* equal to 0.999 and 0.58 day^-1^ as compared to 0.92 and 0.14 day^-1^ with IFN-based therapy, respectively [30]. However this dual mode of action of PIs may be integrated in a more physiological way by using new multiscale viral kinetic models that explicitly integrate the effect of PIs on the intra-cellular viral dynamics [9].

Although the use of NLMEM has been shown to provide very precise and accurate estimates of the parameters even in presence of sparse designs, it should be acknowledged that these estimates are done on the population parameters, *i.e.,* the mean and the variance of parameters in the population. How NLMEM also allow precise and accurate estimation of the individual parameters for individualized treatment duration remains to be evaluated.

## Conclusion

Compared with standard approach (with Wilcoxon test), modeling approach (with Wald test) provides very precise and accurate estimates of viral kinetic parameters and a more powerful tool to detect a difference in early viral kinetic profile of two PIs with different antiviral efficacy, even with sparse initial sampling or small number of patients. When designing a viral kinetic study, our results indicate that the enrollment of a larger number of patients is to be preferred to smaller sample size with more frequent assessments of viral load. We showed that a threshold correction is needed for the Wald test with small samples especially if there are many BLD data.

## Competing interests

All authors declare that they have no competing interests.

## Authors’ contributions

CL, JG and FM designed the simulation study and the MODCUPIC trial. CL carried out the simulations and drafted the manuscript. JG participated to the work of estimation (with CL). CL, JG and FM participated in the statistical analysis and helped to draft the manuscript. All authors read and approved the final manuscript.

## Pre-publication history

The pre-publication history for this paper can be accessed here:

http://www.biomedcentral.com/1471-2288/13/60/prepub
